# The Relationship between Muscular Strength and Depression in Older Adults with Chronic Disease Comorbidity

**DOI:** 10.3390/ijerph17186830

**Published:** 2020-09-18

**Authors:** Jae-Moo Lee, Edward J. Ryan

**Affiliations:** 1 Department of Sport Science, Sungkyunkwan University, Suwon 16419, Korea; ljmmc@skku.edu; 2 Department of Exercise Science, Chatham University, Pittsburgh, PA 15232, USA

**Keywords:** aging, muscular strength, disease comorbidities, depression

## Abstract

Older adults with disease and disability are particularly vulnerable to experiencing more severe consequences of depression. The purpose of the present study is to investigate the relationship between disease comorbidities (number of disease: ND0, 1 disease: ND1 and 2 or more diseases: ND ≥ 2), hand grip strength (low HGS and high HGS), and the prevalence of depression in Korean older adults. Data from the living profiles of older people survey that was conducted by the ministry of health and welfare in Korea were utilized. Data for 6107 females and 4347 males were appropriate for statistical tests. The results demonstrated that depression was more prevalent as ND increased (*p* < 0.01). In addition, HGS appeared lower as ND increased in both male (*p* < 0.01) and female subjects (*p* < 0.01). Furthermore, relative to ND0 and low HGS, ND0 and high HGS showed a ~65% reduction in the prevalence of depression (*p* < 0.01). After adjusting for age, the prevalence of depression was reduced by ~60% in the subgroup with ND0 and high HGS relative to ND0 and low HGS (*p* < 0.01). These data demonstrate that muscular strength indices such as HGS may be useful when assessing depression in older adults. Further research in this area is warranted.

## 1. Introduction

Depression is a mental and emotional disorder that elicits the consistent feelings of sadness, powerlessness and a loss of interest. This disorder can not only interfere with personal daily functioning, but also reduces quality of life in sufferers. The prevalence of depression has risen globally, and approximately 260 million people worldwide suffer from this disorder [1]. In 2017, about 11 million people in the United States (approximately 4.5% of all U.S. adults) had one of the major depressive disorders [2]. Furthermore, chronic mild to severe depression can become a serious health condition, resulting in hospitalization and suicide, triggering a continuous rise in social and medical expenses [3]. Therefore, the World Health Organization (WHO) defines that depression is one of the major health problems. Although the prevalence of depression in the elderly population is relatively less than the younger populations, the physical, psychological and physiological consequences of depression are more severe and can result in fatal outcomes. As such, suicide in older adults is mainly due to depression and the rate is significantly higher than younger age groups [4,5].

The causes of depression may be multifactorial and include a complicated interaction among social, biological, genetic, psychological, socioeconomic and physical factors [5]. However, depression in the elderly population is derived somewhat differently from younger population and in many cases, it is associated with medical illnesses and functional disability [3]. Previous studies found that depression increased in elderly patients with cardiovascular diseases [6,7]. In addition, other studies reported that there was a significant relationship between depression and functional disability in elderly populations, and persistent elevation in depression was observed following the worsening of functional capacity [8,9]. According to the review by Katon, the prevalence of depression in community settings, primary-care settings and inpatient medical settings increases from 3–5% to 5–10% and 10–14%, respectively [10]. This finding suggests that the more severe physical illness older adults have, the more serious depression they suffer. Additionally, Gunn et al. reported that the number of diseases (ND) was closely related to the development of depression [11] and moreover, Kukull et al. demonstrated that ND could be the best predictor of depression in elderly patients with general medical conditions [12]. Therefore, understanding the relationship between disease comorbidities and depression in elderly populations is salient.

In the elderly populations, clinical condition and functional capacity appear to be associated with handgrip strength (HGS), which is a general indicator of muscular strength. Numerous studies observed that lower muscular strength was independently related to development of depression [13,14] and multiple chronic diseases and comorbidities in men and women is inversely related to HGS [15]. Other studies reported that strength training appeared to reduce signs and symptoms of depression [16,17]. Nevertheless, there is a paucity of research investigating how HGS and chronic disease comorbidities influence the prevalence of depression in the elderly population. Therefore, the purpose of the present study is to investigate how HGS influenced depression in male and female older adults with chronic disease comorbidities.

## 2. Methods

### 2.1. Study Population

The present study used data from the living profiles of older people survey (LPOPS) that was conducted by the ministry of health and welfare for the purpose of developing welfare policies and quality of life in elderly populations in Korea. To achieve the purpose of the study, data were initially obtained from 12,344 subjects over the age of 60. However, only data from 10,454 subjects were selected for analysis due to missing data (N = 1890) including chronic disease status, depression, HGS and other variables.

### 2.2. Measurements

#### 2.2.1. Chronic Disease Comorbidities

Chronic disease was defined as one of 34 common chronic diseases based on 5 categories including cardiovascular diseases, diabetes, orthopedic complications, pulmonary diseases and thyroid syndromes, and referred to the diseases that had been diagnosed more than 3 months [18]. For analysis, subjects were divided by 3 groups including a group with no chronic disease, a group with one chronic disease and a group with 2 or more chronic diseases.

#### 2.2.2. Depression

To assess depression, the Short form of Geriatric Depression Scale (SGDS) questionnaire was utilized. The SGDS consists of 15 questions related to depression (mainly focused on mood state) in older adults and it has been considered as an efficient screening tool for clinicians for further assessment [19,20,21]. For the present study, a Korean version of the SGDS developed by Sheikh and Yesavage was utilized [22]. This version demonstrated 0.9429 sensitivity and 0.7255 specificity against the original SGDG [23]. Eight points or higher out of a total 15 points was determined as depression.

#### 2.2.3. Hand Grip Strength

HGS is the force applied by the hand and it has been commonly utilized to predict the overall muscular strength in the elderly population [24,25]. HGS was assessed via the grip strengthener (TKK 5401, Takei Scientific Instruments Co., Ltd., Tokyo, Japan) and was measured twice for both hands and it calculated average values for both hands. Values that were below the bottom 2.5% and above the top 97.5% were considered outliers and excluded from analyses. For analysis, subjects were divided into 2 groups, upper 50% and lower 50%, based on strength in each sex.

#### 2.2.4. Covariate Variables

Data such as basic demographic information including sex, educational background and smoking status, body mass index (BMI), average sleeping hours, cognitive function and physical activity level were also available. Sex consisted of 2 categories (women vs. men), educational background consisted of 4 categories (no education vs. elementary school level vs. high school level vs. college level) and smoking status consisted of 2 categories (never smoked vs. previous and current smoker). In addition, body mass index was calculated by body weight in kg divided by square of height in m2 and cognitive function was measured via Korean version of Mini-Mental State Examination (MMSE-K), which has generally been utilized for the Korean elderly population [26]. Lastly, physical activity level was split into 2 categories based on the WHO guidelines; low physical activity (none or moderate exercise ≤150 min per week) vs. high physical activity (moderate exercise ≥150 min per week or vigorous exercise ≥75 min per week) [27].

#### 2.2.5. Statistical Analysis

Statistical analysis was conducted using SPSS software (IBM, Chicago, IL, USA) (version 24.0). All variables were checked for normality, and if necessary, subjected to log10 transformation before analysis. Continuous variables are presented as means ± standard deviations, and categorical variables reported as percentages. Subjects were divided into three groups for ND. Fitness levels were defined as high or low according to the median values of the sex-specific HGS test. An independent samples *T*-test and the Chi-square test were used to compare the mean differences between variables with respect to sex and HGS. A One-way Analysis of Variance (ANOVA) and Chi-square analyses were used to compare the mean differences between variables in ND-based subgroups.

The combined effects of ND and HGS on the depression were examined using the following 6 subgroups (ND0 with low HGS, ND0 with high HGS, ND1 with low HGS, ND1 with high HGS, ND ≥ 2 with low HGS, ND ≥ 2 with high HGS). Logistic regression analysis was used to estimate risks (OR, odds ratio) of depression for ND and HGS level combinations after adjusting for confounding variables (age, smoking, income, education, MMSE-K and physical activity level). The reference group was the ND 0 with low HGS subgroup (OR = 1). In addition, subsequent analyses were performed for women and men separately. Statistical significance was set a priori at *p* < 0.05.

## 3. Results

### 3.1. General Demographic

For the present study, data were obtained from 6107 female and 4347 male subjects. For disease status, female subjects demonstrated a higher prevalence of disease comorbidities (two or more chronic disease) than male subjects (63.5 vs. 46.0%). In addition, depression was more prevalent in female subjects than male subjects (4.30 ± 4.35 vs. 5.72 ± 4.52, *p* < 0.05). However, female subjects appeared to show significantly lower HGS than male subjects (18.24 ± 4.85 vs. 29.31 ± 7.33, *p* < 0.05). Table 1 illustrates the subject demographic information. Furthermore, female subjects demonstrated higher BMI, lower cognitive function, lower educational level and shorter sleep hours than male subjects (*p* < 0.01). However, there was a higher percentage of previous and current smokers in male subjects than female subjects (*p* < 0.01). Lastly, both male and female subjects demonstrated higher rate of participation in vigorous physical activity than low physical activity.

### 3.2. Diseases Comorbidities, Depression and Grip Strength

The data demonstrated that depression was more prevalent as ND increased (*p* < 0.001, Table 2). In addition, HGS appeared lower as ND increased in both male subjects (*p* < 0.01) and female subjects (*p* < 0.001, Table 2). Moreover, BMI was higher as ND increased (*p* < 0.001), while MMSE score and the amount of sleep were reduced as ND increased (*p* < 0.001, *p* < 0.001, respectively). With respect to physical activity, as ND increased, subjects demonstrated low activity level (*p* < 0.001). Interestingly, older adults with ND ≥ 2 demonstrated a lower percentage of smokers than those with no disease (30.2 vs. 44.4%, *p* < 0.001). Table 2 illustrates the comparison of measured variables according to ND.

Based on HGS (high vs. low), depression was lower in the high HGS group in contrast to the low HGS group (*p* < 0.001, Table 3). In addition, the high HGS group demonstrated lower prevalence of diseases (*p* < 0.001, Table 3). Furthermore, the high HGS group showed higher MMSE score, higher education and higher income (*p* < 0.001, *p* < 0.001, *p* < 0.001, respectively). With respect to physical activity, the high HGS group presented a higher percentage of subjects who were engaged in high activity level than the low HGS group (*p* < 0.001). However, there were no significant differences in smoking history and the amount of sleep between groups (*p* > 0.05, Table 3). Although it was a minor difference, the high HGS group showed higher BMI than the low HGS group (*p* < 0.001, Table 3).

### 3.3. The Combined Effect of Grip Strength and Disease Comorbidities on Depression

Based on the subgroup with ND0 and low HGS (Model 1, Table 4), the subgroup with ND0 and high HGS showed ~65% reduction depression (*p* < 0.001). In addition, while the subgroup with ND1 and low HGS showed ~77% increase in depression (*p* < 0.001), the subgroup with ND 1 and high HGS showed 25% reduction in depression (*p* < 0.001). In the case of ND ≥ 2, while the subgroup with low HGS showed ~3 folds increase in depression (*p* < 0.001), the subgroup with high HGS show only ~58% increase in depression (*p* < 0.001).

After adjusting with age (Model 2, Table 4), depression was reduced by ~60% in the subgroup with ND0 and high HGS relative to subgroup ND0 and low HGS (*p* < 0.001). Based on the subgroup ND0 and low HGS, the subgroup with low HGS showed ~78% increase in depression (*p* < 0.01), but the subgroup with high HGS showed ~15% reduction in depression (*p* < 0.01). In case of ND ≥ 2, the subgroup with low HGS showed higher increase (~3.1 folds) in depression than the subgroup with high HGS (~1.8 folds) (*p* < 0.001). After adjusting with covariate variables including age (Model 2, Table 4) and education, smoking, BMI, MMSE, sleep time and physical activity (Model 3, Table 4), the results remained similar.

In men, depression was reduced by ~60% in the subgroup with ND0 and high HGS relative to subgroup ND0 and low HGS (Model 1, Table 4, *p* < 0.001). Furthermore, the subgroup with ND1 and low HGS demonstrated ~2 folds increase in depression while the subgroup with ND1 and high HGS showed ~20% reduction in depression (Model 1, Table 4, *p*<0.001). In the case of ND ≥ 2, the subgroup with low HGS presented ~3.5 folds increase in depression; however, the subgroup with high HGS presented only ~13% increase in depression (Model 1, Table 4, *p* < 0.001). In the women group, based on the subgroup with ND0 and low HGS, the subgroup with ND0 and high HGS showed ~70% reduction in depression. In addition, while depression was increased by ~43% and ~2.3 folds in the subgroups with ND1 and low HGS (*p* < 0.001) and ND2 and low HGS (*p* < 0.001), respectively, it was reduced by ~35% in the subgroup with ND1 and high HGS (*p* < 0.001) and increased by only ~30% in the subgroup with ND2 with high HGS (*p* < 0.001). Both men and women presented similar phenomenon after adjusting for age (Model 2, Table 4) and other variables including cognitive function, BMI, sleep time, smoking, education, income and physical activity (Model 3, Table 4).

## 4. Discussion

The present study utilized a large cohort data set to investigate how HGS and ND impact the prevalence of depression in the Korean elderly population. These data demonstrated depression became more prevalent as ND increased and the prevalence of depression appeared low when HGS was greater. Furthermore, the results indicated that at a given ND, the group with greater HGS demonstrated a much lower score of depression. This finding indicates that HGS may be a useful measurement when assessing depression in the elderly population.

Generally, the risk of depression becomes greater with age [28] and chronic disease can impact depression independent of age [29]. However, chronic diseases seem to worsen depression in the elderly population. A study on the Australian elderly population found that depression was positively associated with ND [11]. This result is accordant to our finding from the Korean elderly population. In addition, a similar result was observed in a meta-analysis by Read et al. [30], revealing that the group with 1 chronic disease had a 2-fold higher risk of depression than the group with the absent of chronic disease, and the group with two or more chronic diseases had 3 folds higher risk. As such, chronic disease may be a salient factor for depression in the elderly.

The present finding regarding HGS and depression was similar to the results from other previous studies. Muscular strength has often been defined as having a relationship with mental and mood health [31,32]. Likewise, the reduction in HGS, well representing overall muscular strength, was connected to the deterioration of depression [13] since a reduced muscular strength can limit activity daily living (ADL) and mobility in the elderly and this may elicit depression [33]. This is supported by a longitudinal study on the Irish elderly, reporting the inverse relationship between HGS and depression [34]. Furthermore, this inverse relationship was more pronounced in the women group [34]. However, there are also some conflicting results regarding a relationship between HGS and depression. According to Stessman et al. [35], HGS could be a predicting factor for the prevalence of diseases and mortality in the elderly, but not depression. In addition, a study by Kweon et al. observed that lower HGS was not related to depression in the elderly aged 40–79 years [36]. This discrepancy has not been clearly defined yet. However, it can be speculated that these conflicting outcomes may result from different study populations and characteristics such as age, nationality and disease status.

Chronic disease and HGS have an inverse relationship in the elderly and HGS may be a more significant predictor for chronic disease than chronological age [15,37]. Aforementioned, chronic disease and HGS had contrasting effects on depression in the present study. However, it must be noted how a combination of chronic disease and HGS influence depression. In all groups with ND0, ND1 and ND ≥ 2, a group with higher HGS demonstrated lower depression relative to a group with lower HGS. In addition, the phenomenon that depression becomes more severe as ND increases can be altered by higher or lower HGS. For example, the subgroup with ND1 and high HGS 1 showed lower depression than the subgroup with N0 and low HGS [OR (95% CI): 0.749 (0.614–0.913) vs 1.00]. After adjusting for age (Model 2, Table 4), depression in all groups with ND0, ND1 and ND ≥ 2 was still lower for high HGS relative to low HGS. Additionally, this finding remained unchanged after adjusted for age, sex, education level, smoking, BMI, cognitive function and sleep time (Model 3, Table 4). These findings suggest that the relationship between depression and chronic disease comorbidities can be changed by HGS and it emphasizes how important maintenance of muscular strength in the elderly is against depression following chronic diseases.

The phenomenon that HGS was significantly associated with depression irrespective of disease comorbidities was present regardless of sex. Moreover, the results remained unchanged after adjusted covariate variables in both men and women. However, overall, it seems that men demonstrated a greater difference in depression between low HGS and high HGS as ND increased when comparing to women. In addition, with low HGS, men presented a more pronounced increase in depression as ND increased relative to women and this result was similar across Models 1, 2 and 3. However, outcomes were relatively similar between men and women in the case of high HGS. Generally, it is believed that depression is more prevalent in women than men [38,39] and in the present study consistent results are observed. However, there is no clear explanation of why men demonstrate a higher prevalence as ND increases when HGS is low in this study. Previous research has demonstrated that perceived health status was a significant predictor of depression in older adults [40]. Therefore, it is possible that in this study, men are more sensitive to ND to have depression when HGS is low. However, further research is needed to confirm this sex-related impact on depression along with ND.

The present study has some limitations. Although this study included a large cohort sample of the Korean elderly population, these results may not be generalizable to other ethnic groups and/or countries. The present study utilized a self-report system to assess the number of chronic diseases. Due to the nature of this system, there could be a bias that exaggerates the results. In addition, this self-report system did not acquire the severity of diseases and this could influence the results. Depression and cognitive function were assessed via single measurements. Lastly, the present study did not include factors known to influence depression, such as seasonal health condition and marital status.

## 5. Conclusions

In conclusion, these results demonstrate that muscular strength indices such as HGS may be useful when assessing depression in older adults. Further studies are needed to elucidate the complicated interaction between factors that influence the prevalence and severity of depression in older adults.

## Figures and Tables

**Table 1:** Subject characteristics.

Baseline Characteristics of Study Participants				
**Variables**	**Category**	**Men**	**Women**	**Total**
		**N = 4347**	**N = 6107**	**N = 10,454**
		**mean ± SD**	**mean ± SD**	**mean ± SD**
Age (years)		72.40 ± 5.65	73.12 ± 5.92	2.82 ± 5.82
Depression (score)		4.30 ± 4.35	5.72 ± 4.52	5.13 ± 4.46
MMSE-K (score)		24.86 ± 3.74	22.03 ± 4.44	23.21 ± 4.39
BMI (Kg/m^2^)		23.02 ± 2.99	23.91 ± 3.46	23.54 ± 3.31
Sleep time (minutes)		409.40 ± 96.19	381.76 ± 94.48	393.25 ± 96.16
Hand-grip strength (Kg)		29.31 ± 7.33	18.24 ± 4.85	22.84 ± 8.12
Income (10.000 won)		144.15 ± 165.98	122.45 ± 155.79	131.477 ± 160.46
		**N (%)** **N = 4347**	**N (%)** **N = 6107**	**N (%)** **N = 10,454**
Physical activity	High activity level	1134 (21.6)	782 (12.8)	1916 (18.3)
	Low activity level	3213 (73.9)	5325 (87.2)	8538 (81.7)
Ex/current smoking		3220 (74.1)	464 (7.6)	3684 (35.2)
Education	None	651 (15.0)	2965 (48.6)	3616 (34.6)
	Elementary	1743 (40.1)	2366 (38.7)	4109 (39.3)
	Middle & High	1471 (33.8)	688 (11.3)	2159 (20.7)
	≥College	482 (11.1)	88 (1.4)	570 (5.5)

**Table 2:** Comparisons of variables following the number of chronic diseases.

	Number of Diseases				
**N**	**0** **N = 1863**	**1** **N = 2715**	**2** **N = 5876**	***p* Value**	**Post-Hoc**
	**mean ± SD**	**mean ± SD**	**mean ± SD**		
Depression(score)	3.45 ± 3.71	4.42 ± 4.23	5.99 ± 4.58	<0.001	a < b < c
Handgrip strength (kg)	25.73 ± 8.40	23.97 ± 8.33	21.41 ± 7.58	<0.001	a > b > c
Men	30.93 ± 6.85	29.80 ± 7.22	28.15 ± 7.33	<0.001	a > b > c
Women	19.21 ± 4.88	18.52 ± 4.92	17.93 ± 4.79	<0.001	a > b > c
MMSE-KC (score)	24.11 ± 4.38	23.47 ± 4.57	22.81 ± 4.25	<0.001	a > b > c
BMI (Kg/m^2^)	22.78 ± 2.97	23.28 ± 3.18	23.90 ± 3.41	<0.001	a < b < c
Sleep time (minutes)	407.38 ± 92.76	395.63 ± 94.46	386.68 ± 97.51	<0.001	a < b < c
Income (10.000 won)	146.23 ± 176.01	136.09 ± 187.02	124.61 ± 140.38	<0.001	a, b < c
	**N (%)** **N = 1863**	**N (%)** **N = 2715**	**N (%)** **N = 5876**		
Physical activity					
High activity level	451 (24.2)	542 (20.0)	923 (15.7)	<0.001	
Low activity level	1412 (75.8)	2173 (80.0)	4953 (84.3)		
Smoking, N (%)					
Ex/current smoking	828 (44.4)	1081 (39.8)	1775 (30.2)	<0.001	
None	1035 (55.6)	1634 (60.2)	4101 (69.8)		
Education, N (%)					
None	530 (28.4)	889 (32.7)	2197 (37.4)	<0.001	
Elementary	718 (38.5)	1081 (39.8)	2310 (39.3)		
Middle and High	464 (24.9)	588 (21.7)	1107 (18.8)		
College	151 (8.1)	157 (5.8)	262 (4.4)		

**Table 3:** Comparison of variables following handgrip strength.

	Handgrip Strength		
**N**	**Low** **N = 4981**	**High** **N = 5473**	***p* Value**
	**mean ± SD**	**mean ± SD**	
Depression (score)	6.26 ± 4.62	4.10 ± 4.04	<0.001
Number of Disease, n (%)			
None	700 (14,1)	1161 (21.2)	<0.001
one	1223 (24.6)	1492 (27.3)	
≥Two	3059 (61.4)	2818 (51.5)	
MMSE-KC (score)	21.91 ± 4.68	24.40 ± 3.72	<0.001
BMI (Kg/m^2^)	23.13 ± 3.45	23.92 ± 3.12	<0.001
Sleep time (minutes)	391.58 ± 101.18	394.78 ± 91.33	0.89
Income (10.000 won)	12.117 ± 147.75	140.85 ± 170.68	<0.001
	**N (%)** **N = 4981**	**N (%)** **N = 5473**	
Physical activity			
High activity level	649 (13.0)	1267 (23.2)	<0.001
Low activity level	4332 (87.0)	4206 (76.8)	
Smoking, N (%)			
Ex/current smoking	1727 (34.7)	1957 (35.8)	0.251
None	3252 (65.3)	3516 (64.2)	
Education, N (%)			
None	2113 (42.3)	1503 (27.5)	<0.001
Elementary	1892 (38.0)	2217 (40.5)	
Middle and High	779 (15.6)	1380 (25.2)	
College	197 (4.0)	373 (6.8)	

**Table 4:** Combined effects of handgrip strength and the number of diseases on depression.

Sex	Exposures		Model 1	*p* Value	Model 2	*p* Value	Model 3	*p* Value
	ND	HGS	OR ^1^ (95%CI)		OR ^2^ (95%CI)		OR ^3^ (95%CI)	
**Total**	0	low	1		1		1	
	0	high	0.356 (0.274–0.461)	<0.001	0.405 (0.312–0.527)	<0.001	0.519 (0.395–0.682)	<0.001
	1	low	1.765 (1.448–2.130)	<0.001	1.782 (1.448–2.164)	<0.001	1.663 (1.355–2.041)	<0.001
	1	high	0.749 (0.614–0.913)	<0.001	0.844 (0.690–1.033)	<0.001	1.016 (0.821–1.258)	<0.001
	≥2	low	2.990 (2.454–3.642)	<0.001	3.071 (2.518–3.746)	<0.001	2.878 (2.331–3.554)	<0.001
	≥2	high	1.575 (1.282–1.935)	<0.001	1.764 (1.431–2.174)	<0.001	1.899 (1.519–2.373)	<0.001
**Men**	0	low	1		1		1	
	0	high	0.399 (0.272–0.587)	<0.001	0.442 (0.300–0.652)	<0.001	0.624 (0.416–0.937)	<0.001
	1	low	2.036 (1.523–2.723)	<0.001	2.029 (1.516–2.714)	<0.001	2.076 (1.525–2.828)	<0.001
	1	high	0.793 (0.585–1.074)	<0.001	0.866 (0.637–1.178)	<0.001	1.230 (0.885–1.708)	<0.001
	≥2	low	3.588 (2.630–4.895)	<0.001	3.520 (2.578–4.805)	<0.001	3.710 (2.663–5.168)	<0.001
	≥2	high	1.1319 (0.930–1.871)	<0.001	1.428 (1.004–2.032)	<.001	2.003 (1.373–2.923)	<0.001
**Women**	0	low	1		1		1	
	0	high	0.318 (0.223–0.454)	<0.001	0.367 (0.256–0.527)	<0.001	0.435 (0.299–0.633)	<0.001
	1	low	1.429 (1.098–1.859)	<0.001	1.487 (1.141–1.937)	<0.001	1.365 (1.033–1.803)	<0.001
	1	high	0.640 (0.490–0.837)	<0.001	0.737 (0.561–0.969)	<0.001	0.846 (0.634–1.128)	<0.001
	≥2	low	2.249 (1.727–2.930)	<0.001	2.405 (1.842–3.140)	<0.001	2.289 (1.728–3.031)	<0.001
	≥2	high	1.318 (1.005–1.728)	<0.001	1.516 (1.150–2.000)	<0.001	1.587 (1.186–2.125)	<0.001

Statistical models are as follow: Model 1: no adjustment; Model 2 adjusted for age; Model 3: adjusted for body mass index (BMI), income, physical activity, education levels, smoking and sleep time.
